# Association of miR-548c-5p, miR-7-5p, miR-210-3p, miR-128-3p with recurrence in systemically untreated breast cancer

**DOI:** 10.18632/oncotarget.24088

**Published:** 2018-01-09

**Authors:** Ines Block, Mark Burton, Kristina P. Sørensen, Lars Andersen, Martin J. Larsen, Martin Bak, Søren Cold, Mads Thomassen, Qihua Tan, Torben A. Kruse

**Affiliations:** ^1^ Department of Clinical Genetics, Odense University Hospital, Odense, Denmark; ^2^ Human Genetics, Department of Clinical Research, University of Southern Denmark, Odense, Denmark; ^3^ Department of Pathology, Odense University Hospital, Odense, Denmark; ^4^ Department of Oncology, Odense University Hospital, Odense, Denmark; ^5^ Epidemiology, Department of Public Health, University of Southern Denmark, Odense, Denmark

**Keywords:** low-risk breast cancer, microRNA, prognosis, lymph node negative, estrogen receptor positive

## Abstract

Current prognostic markers allocate the majority of lymph node (LN) negative and estrogen receptor (ER) positive breast cancer patients into the high-risk group. Accordingly, most patients receive systemic treatments although approximately 40% of these patients may have been cured by surgery and radiotherapy alone. Two studies identified seven prognostic microRNAs in systemically untreated, LN negative and ER positive breast cancer patients which may allow more precise patient classification. However, six of the seven microRNAs were analyzed in both studies but only found to be prognostic in one study. To validate their prognostic potential, we analyzed microRNA expression in an independent cohort (*n* = 110) using a pair-matched study design minimizing dependence of classical markers. The expression of hsa-miR-548c-5p was significantly associated with abridged disease-free survival (hazard ratio [HR]:1.96, *p* = 0.027). Contradicting published results, high hsa-miR-516-3p expression was associated with favorable outcome (HR:0.29, *p* = 0.0068). The association is probably time-dependent indicating later relapse. Additionally, re-analysis of previously published expression data of two matching cohorts (*n* = 100, *n* = 255) supports an association of hsa-miR-128-3p with shortened disease-free survival (HR:2.48, *p* = 0.0033) and an upregulation of miR-7-5p (*p* = 0.0038; *p* = 0.039) and miR-210-3p (*p* = 0.031) in primary tumors of patients who experienced metastases. Further analysis may verify the prognostic potential of these microRNAs.

## INTRODUCTION

Breast cancer is the most frequent cancer in women worldwide and is associated with a high mortality rate of approximately 15% despite recent improvements in diagnosis and treatment [1, 2]. The main cause of death is not the primary tumor in the breast, but the occurrence of lethal metastases in essential organs like lung, liver and the brain. Lymph node (LN) negative breast cancer patients do not present any signs of distant metastasis at the time of diagnosis. However, clinical and histopathological criteria currently classify the majority of LN negative and estrogen receptor (ER) positive patients as high-risk patients who accordingly receive adjuvant systemic treatments after surgical removal of the primary tumor [2]. In fact, approximately 40% of these patients do not benefit from the systemic treatment and would be cured by the removal of the primary tumor and radiotherapy alone [3, 4]. Consequently, improved prognostic markers are required to prevent adverse side-effects in systemically treated patients and to reduce health care costs.

Larger studies were conducted to establish various multi-analyte tests like MammaPrint, Oncotype DX, Breast Cancer Index, EndoPredict or Prosigna (PAM50) to improve outcome prediction and stratify therapy selection in breast cancer patients [5, 6]. Nevertheless, comparable studies among LN negative and ER positive patients to reduce systemic treatment are rare, most likely because only limited sample material is available. Fresh frozen tissue samples are preferred for higher technical quality and long follow-up times are required to determine which patients will or will not experience metastases 10, 15 or 20 years post diagnosis. Moreover, patients should not have received any systemic treatment, because markers developed based on treated cohorts could not distinguish between the future absence of metastases and positive treatment response. Due to today’s unspecific classification of patients, the collection of suitable sample sets in a critical scale is nowadays challenging. Esserman *et al.* used Formalin-Fixed, Paraffin-Embedded tissue samples collected in Sweden more than 20 years ago. They adjusted the mRNA expression-based MammaPrint cut-off to identify a smaller group of so-called ultralow-risk patients who may not benefit from any adjuvant systemic treatment [7]. However, better prognostic markers are required to substantially increase the ultralow patient group and to ensure that the rate of recurrence will not increase as a consequence of treatment reduction.

In the past decades microRNAs (miRs) have intensively been studied in primary breast tumor tissue and a few prognostic miRs have been proposed [8–10]. Although miRs are considered as key-regulators of the breast cancer transcriptome, none of the so far identified prognostic miRs have, to our knowledge, passed larger clinical trials [11]. Bias concerning the analyzed sample material, patient cohort heterogeneity as well as methodological and analytical variation limits the initial validation of candidate miRs. Lánczky *et al*. recently succeeded in validating the predictive potential of 26 out of 41 selected miRs by analyzing e.g. subtype- or receptor-specific patient cohorts with the integrated platform miRpower in a large group of mainly systemically treated patients [12]. In comparison, two studies focused solely on the *de novo* identification of prognostic miRs in primary tumor samples of LN negative, ER positive patients who did not receive any systemic treatment. Foekens *et al.* analyzed 249 miRs in 37 primary tumor samples via qRT-PCR and validated four prognostic miRs (hsa-miR-210-3p, hsa-miR-7-5p, hsa-miR-128-3p, hsa-miR-516-3p) by analyzing 147 additional tumor samples [13]. D’Aiuto *et al*. selected erb-b2 receptor tyrosine kinase 2 (ERBB2) negative samples (*n* = 92) and identified hsa-miR-548c-5p, hsa-miR-30e-3p and hsa-miR-125b-5p as differentially expressed in patients with and without relapse [14]. D’Aiuto *et al*. further validated the prognostic potential of hsa-miR-30e-3p (hsa-miR-30e*; MIMAT0000693) in an independent cohort of 223 matching samples extracted from the Molecular Taxonomy of Breast Cancer International Consortium (Metabric) data set [15, 16]. Although, both studies independently analyzed the expression of six out of the seven candidate miRs, each study identified an individual set of prognostic miRs. Considering this inconsistency, we aimed at cross-validating the suggested prognostic miRs to potentially identify those who could stratify classification of LN negative and ER positive patients in the future. Firstly, we collected 55 fresh frozen primary tumors from systemically untreated patients who developed distant metastasis and matching 55 fresh frozen primary tumors from patients who remained metastasis-free. We used a paired study design for increased statistical power and to analyze the differential expression of the candidate miRs and their association with outcome independently of any confounding prognostic factors [17, 18]. Moreover, we re-analyzed results presented by D’Aiuto *et al.* including ERBB2 positive patients and additionally analyzed candidate miRs identified by Foekens *et al.* in the independent patient cohorts published by D’Aiuto *et al.* and Metabric [14–16].

## RESULTS

D’Aiuto *et al.* identified hsa-miR-1308, hsa-miR-548c-5p (MIMAT0004806), hsa-miR-125b-5p (MIMAT0000423) and hsa-miR-30e-3p as differentially expressed (*p* < 0.001) in LN negative, ER positive and ERBB2 negative breast cancer patients [14, 19]. The authors confirmed the prognostic role of hsa-miR-30e-3p in a matching cohort of 223 untreated patients extracted from the Metabric cohort [15, 16]. We aimed at validating the candidate miRs (except for hsa-miR-1308 which was later identified as being part of a t-RNA) [19] in our independent LN negative, ER positive cohort using a paired study design. To match our patient characteristics and analysis parameters, we further re-analyzed the role of these miRs in the D’Aiuto *et al.* and Metabric cohorts (Table 1) but included ERBB2 positive samples and divided the samples into high/low expression groups as being above or below the median expression value.

**Table 1:** Patient and tumor characteristics of the 4 cohorts of systemically untreated, lymph node negative and estrogen receptor positive breast cancer patients included in the study

	OUH (*n* = 110)		D’Aiuto *et al.* (*n* = 100)		Metabric (*n* = 255)		Foekens *et al.* (*n* = 147)^**^	
	No. of patients (%)							
Metastasis	yes	no	yes	no	yes	no	yes	no
	55 (50.0%)	55 (50.0%)	47 (47.0%)	53 (53.0%)	65 (25.5%)	190 (74.5%)	n/a	n/a
**Age at diagnosis**								
≤50 years	10 (9.1%)	9 (8.2%)	12 (12.0%)	22 (22.0%)	22 (8.6%)	49 (19.2%)	79 (53.7%)	
>50 years	45 (40.9%)	46 (41.8%)	35 (35%)	31 (31%)	43 (16.9%)	141 (55.3%)	68 (46.3%)	
Tumor size								
≤ 2 cm	23 (20.9%)	23 (20.9%)	21 (21.0%)	34 (34.0)	36 (14.1%)	118 (46.3%)	82 (55.8%)	
2–5 cm	32 (29.1%)	32 (29.1%)	24 (24.0%)	19 (19.0)	26 (10.2%)	62 (24.3%)	65 (44.2%)	
>5 cm			1 (1.0%)		2 (0.8%)	6 (2.4%)		
n/a			1 (1.0%)		1 (0.4%)	4 (1.6%)		
Estrogen receptor status^*^								
Positive	49 (44.5%)	48 (43,6%)	47 (47.0%)	53 (53.0%)	62 (24.3%)	173 (67.8%)	147 (100%)	
Negative	1 (0.9%)	4 (3.6%)			1 (0.4%)	9 (3.5%)		
n/a	5 (4.5%)	3 (2.7%)			2 (0.8%)	8 (3.1%)		
**Tumor type**								
Invasive ductal carcinoma	41 (37.3%)	42 (38.2%)	37 (37.0%)	38 (38.0%)	50 (19.6%)	125 (49.0%)		
Invasive lobular carcinoma	9 (8.2%)	9 (8.2%)	9 (9.0%)	3 (3.0%)	7 (2.7%)	15 (5.9%)		
Mucinous carcinoma	2 (1.8%)	2 (1.8%)			1 (0.4%)	9 (3.5%)		
Papillary carcinoma	2 (1.8%)	1 (0.9%)						
Carcinoma with metaplasia	1 (0.9%)	1 (0.9%)						
mixed IDC/ILC			1 (1.0%)	5 (5.0%)	3 (1.2%)	7 (2.7%)		
other				7 (7.0%)	4 (1.6%)	31 (12.2%)		
n/a						3 (1.2%)	147 (100%)	
**Histologic grade**								
1 (good)	12 (10.9%)	15 (13.6%)			7 (2.7%)	33 (12.9%)	29 (19.7%)	
2 (intermediate)	22 (20.0%)	19 (17.3%)			41 (16.1%)	103 (40.4%)		
3 (poor)	7 (6.4%)	7 (6.4%)			12 (4.7%)	40 (15.7%)	71 (48.3%)	
n/a	14 (12.7%)	14 (12.7%)			5 (2.0%)	14 (5.5%)	47 (32.0%)	
**Median year of surgery**	1980–1999							
	1993	1992	n/a	n/a	n/a	n/a	n/a	n/a
**Mean time to metastasis (months)**								
	57.2	n/a	29.8^†^	n/a	n/a	n/a	<60	n/a
**Mean follow up (months)**								
	102.8	238.3	n/a	119.1^†^	108.9	133.6	84 (displayed in graph^**^)	

^*^ as defined by immunohistochemistry (IHC); ^**^patient data of validation analysis [13]; ^†^disease free survival; n/a: not available/applicable; IDC: invasive ductal carcinoma; ILC: invasive lobular carcinoma.

**Figure 1:**
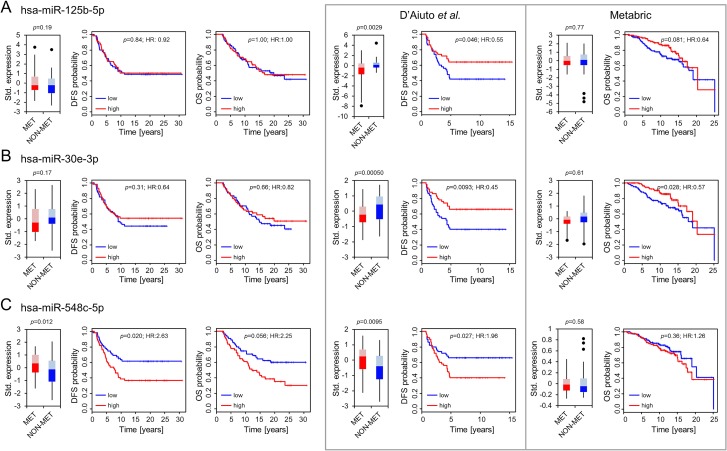
Differential expression and association with outcome for hsa-miR-125b-5p (**A**), hsa-miR-30e-3p (**B**) and hsa-miR-548c-5p (**C**). Boxplots present differences in expression patterns between patients who developed later metastasis (MET) and those who did not (NON-MET). Whiskers correspond to the upper and lower 25% range, data points deviating more than 3*STDEV from the mean are displayed as dots. Lighter colors indicate the 50–75 percentile range, while darker colors indicate the 25–50 percentile range of miRs expressed in MET (red) and NON-MET (blue). Significance was calculated using a paired Student’s *T*-test for the OUH cohort (left panel) and an unpaired Student’s *T*-test for the D’Aiuto *et al*. and Metabric cohort. Kaplan Meier plots indicating disease free (DFS) and overall survival (OS) probabilities were constructed using a univariable COX regression model. Results from the OUH cohort (*n* = 110) are displayed in line with re-analyzed results for the D’Aiuto *et al*. (*n* = 100) and the Metabric (*n* = 255) cohort (encased in grey) as published previously [14].

While we could not confirm a significant expression difference of hsa-miR-125b-5p or hsa-miR-30e-3p (Figure 1A and 1B, Table 2) we observed a trend showing a moderate upregulation of miR-30e-3p in primary tumors of patients who did not develop metastasis (*p* = 0.17). The same trend is also reflected by the survival curves considering disease-free and overall survival (Figure 1B and Table 2; hazard ratio [HR]:0.64, *p* = 0.31 and HR:0.82, *p* = 0.66 respectively). In comparison, a significant expression upregulation in patients with relapse could be demonstrated for hsa-miR-548c-5p (*p* = 0.012; Figure 1C). Furthermore, high hsa-miR-548c-5p expression was associated with abridged disease-free survival (HR: 2.63, *p* = 0.020) and tended to result in shorter overall survival (HR:2.25, *p* = 0.056) (Figure 1C, Table 2). However, the results could not be reproduced in the large Metabric cohort (Figure 1C, Table 2). Potential reasons may be a more heterogeneous cohort composition with shorter follow up, varying sample treatment or a possible cross-reactivity of the applied array format (customized Agilent array) [20] considering that hsa-miR-548c-5p belongs to a rather large miR family comprising 74 human miRs with similar sequences [19]. Although the Metabric cohort comprises more patients, the statistical power is not fundamentally superior in comparison to the other cohorts because of the unbalanced distribution of patients with (*n* = 65) and without (*n* = 190) recurrence.

**Table 2:** Overview summarizing expression and survival analysis for miRs identified by D’Aiuto *et al*. [14]

		D’Aiuto *et al*.^*^ identification	Metabric^*^ 1st validation	OUH 2nd validation
**hsa-miR-125b-5p**				
Diff. Exp.	*p*-value	down in MET	0.77	0.19
		0.0015		
DFS	*p*-value	0.018	-	0.84
	HR (CI)	0.47 (0.21–0.75)	-	0.92 (0.42–2.02)
OS	*p*-value	-	0.081	1.00
	HR (CI)	-	0.64 (0.39–1.06)	1.00 (0.43–2.31)
**hsa-miR-30e-3p**				
Diff. Exp.	*p*-value	down in MET	0.61	0.17
		0.0005		
DFS	*p*-value	0.0094	-	0.31
	HR (CI)	0.45 (0.25–0.82)	-	0.64 (0.28–1.49)
OS	*p*-value	-	0.028	0.66
	HR (CI)	-	0.57 (0.34–0.94)	0.82 (0.34–1.97)
**hsa-miR-548c-5p**				
Diff. Exp.	*p*-value	up in MET	0.58	up in MET
		0.010		0.012
DFS	*p*-value	0.027	-	0.02
	HR (CI)	1.96 (1.08–3.56)	-	2.63 (1.16–5.93)
OS	*p*-value	-	0.36	0.056
	HR (CI)	-	1.26 (0.77–2.06)	2.25 (0.98–5.18)

^*^ re-evaluation of already published results [14]; Diff. Exp.: Differential expression; MET: primary tumors from patients with relapse; HR: Hazard ratio; CI: 95% Confidence interval.

**Figure 2:**
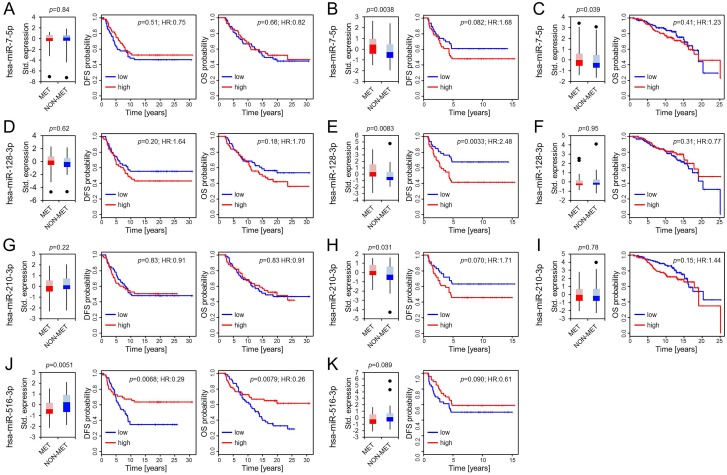
Differential expression of hsa-miR-7-5p, hsa-miR-128-3p, hsa-miR-210-3p and hsa-miR-516-3p Boxplots present differences in expression patterns between patients who developed later metastasis (MET) and those who did not (NON-MET) from the OUH, the D’Aiuto *et al*. and the Metabric cohort (panels from left to right). Whiskers correspond to the upper and lower 25% range, data points deviating more than 3*STDEV from the mean are displayed as dots. Lighter colors indicate the 50–75 percentile range while darker colors indicate the 25–50 percentile range of miRs expressed in MET (red) and NON-MET (blue). Significance was calculated using a paired Student’s *T*-test for the OUH cohort (**A**, **D**, **G**, **J**) and an unpaired Student’s *T*-test for the D’Aiuto *et al.* (**B**, **E**, **H**, **K**) and Metabric cohort (**C**, **F**, **I**). Kaplan Meier plots display disease free (DFS) and overall survival (OS) along with the univariate COX-PH regression significance levels.

Foekens *et al.* identified four miRs in 37 LN negative, ER positive primary breast tumor samples and validated their association with reduced DFS when highly expressed in further 147 LN negative, ER positive primary tumors [13]. We evaluated the differential expression of these miRs in our matching data set but could neither confirm a differential expression of hsa-miR-7-5p (MIMAT0000252), hsa-miR-128-3p (MIMAT0000424) and hsa-miR-210-3p (MIMAT0000267), nor a specific association with abridged disease-free or overall survival (Figure 2A, 2D, 2G; Table 3) [19]. Furthermore, opposing Foekens *et al.* results, we found hsa-miR-516-3p (hsa-miR-516a-3p; hsa-miR-516b-3p; MIMAT0002860; MIMAT0006778) [19] significantly downregulated in primary tumors of patients who developed later metastasis accompanied by a significant association of low hsa-miR-516-3p expression with metastasis formation (HR:0.29, *p* = 0.0068) and shorter overall survival (HR: 0.26, *p* = 0.0079; Figure 2J).

**Table 3:** Overview summarizing expression and survival analysis for miRs identified by Foekens *et al.* [13]

		Foekens et al.^*^ identification	OUH validation	D’Aiuto *et al.* validation	Metabric validation
**hsa-miR-7-5p**					
Diff. Exp.	*p*-value	up in MET	0.83	up in MET	up in MET
				0.0038	0.039
DFS	*p*-value	≤0.05	0.51	0.082	-
	HR (CI)	>1	0.75 (0.32–1.78)	1.68 (0.94–3.01)	-
OS	*p*-value	-	0.66	-	0.41
	HR (CI)	-	0.82 (0.34–1.97)	-	1.23 (0.75–2.02)
**hsa-miR-128-3p**					
Diff. Exp.	*p*-value	up in MET	0.61	up in MET	0.95
				0.008	
DFS	*p*-value	≤0.05	0.20	0.0033	-
	HR (CI)	>1	1.64 (0.77–3.47)	2.48 (1.35–4.55)	-
OS	*p*-value	-	0.18	-	0.31
	HR (CI)	-	1.70 (0.78–3.71)	-	0.77 (0.47–1.27)
**hsa-miR-210-3p**					
Diff. Exp.	*p*-value	up in MET	0.22	up in MET	0.78
				0.031	
DFS	*p*-value	≤0.05	0.83	0.070	-
	HR (CI)	>1	0.91 (0.39–2.14)	1.71 (0.96–3.07)	-
OS	*p*-value	-	0.83	-	0.15
	HR (CI)	-	0.91 (0.39–2.14)	-	1.44 (0.88–2.35)
**hsa-miR-516-3p**					
Diff. Exp.	*p*-value	up in MET	down in MET	0.089	-
			0.0051		
DFS	*p*-value	≤0.05	0.0068	0.090	-
	HR (CI)	>1	0.29 (0.12–0.71)	0.61 (0.34–1.08)	-
OS	*p*-value	-	0.0079	-	-
	HR (CI)	-	0.26 (0.098–0.70)	-	-

^*^ according to published results [13]; Diff. Exp.: Differential expression; MET: primary tumors from patients with relapse; HR: Hazard ratio; CI: 95% Confidence interval.

**Figure 3:**
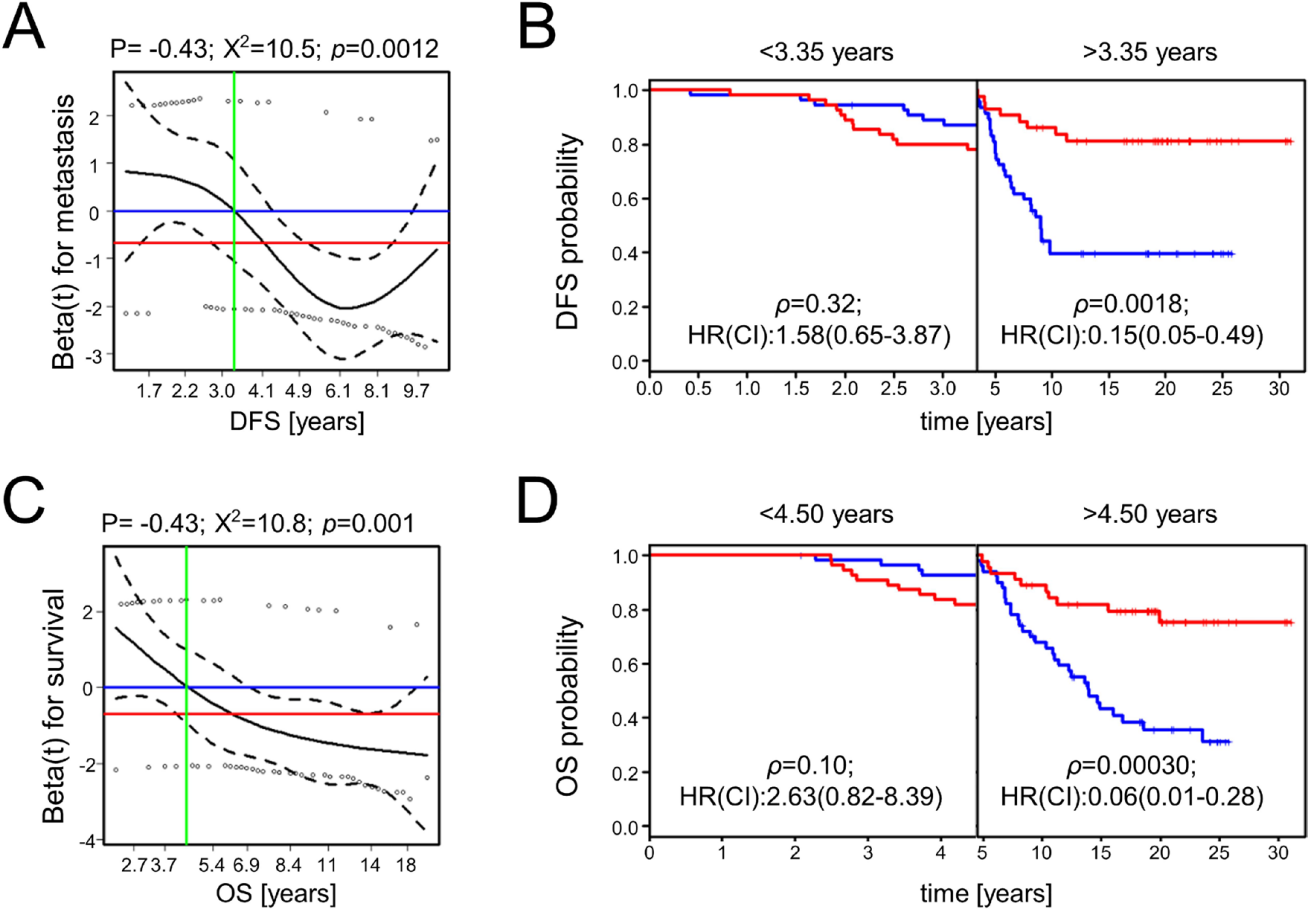
Scaled Schoenfeld residuals for hsa-miR-516-3p and survival analysis using two time intervals The scaled Schoenfeld residuals (**A**, **C**) are shown by circles. The black line is the smoothed mean of the scaled Schoenfeld residuals while the corresponding 95% confidence interval is indicated by the dashed lines. The blue horizontal line corresponds to a hazard ratio of one, and the red horizontal line is the hazard ratio assessed by the univariable COX proportional hazard ratio model (0.29 and 0.26 for DFS and OS, respectively; Figure 2J). The vertical green line shows the time point upon which the hazard ratio shifts from a value >1 to a value <1, representing the time point when the prediction association changes. These time points were used in a subsequent landmark analysis to define two time intervals for disease-free (**B**) and overall survival analysis (**D**) in which the proportional hazard assumption was fulfilled.

To further clarify the potential role of these four miR candidates, we additionally analyzed their expression and outcome association in the available D’Aiuto *et al.* and Metabric data sets (Figure 2; Table 3) [14–16]. A differential expression of hsa-miR-7-5p was found in the D’Aiuto *et al.* (*p* = 0.0038; Figure 2B) and the Metabric cohort (*p* = 0.039; Figure 2C) supporting Foekens *et al.* initial results. However, no significant association with disease free survival (DFS) or overall survival was detected. For hsa-miR-128-3p a prognostic role could be determined in the D’Aiuto *et al.* set (Figure 2E) where a high expression was significantly associated with poor outcome (HR:2.48, *p* = 0.0033). No significant association was computed for hsa-miR-128-3p in the Metabric cohort (Figure 2F). Foekens *et al.* demonstrated a prognostic role for hsa-miR-210-3p in ER positive and negative breast cancer, while the latter was not in the scope of this study, a significant correlation between outcome and hsa-miR-210-3p expression in ER positive patients could not be validated (Figure 2G, 2H, 2I; Table 3) although expression was significantly upregulated in samples from patients with relapse in the D’Aiuto *et al.* data set (Figure 2H). Hsa-miR-516-3p was not analyzed in the Metabric study and data available for the D’Aiuto *et al.* cohort did not confirm an advantageous low or high expression although a trend towards a beneficial low expression could be observed (HR:0.61, *p* = 0.090; Figure 2K, Table 3). Methodological differences and variable cohort features could explain the bias between Foekens *et al.* and our results. For example, in comparison to the D’Aiuto *et al.* and the Foekens *et al.* data sets, we could grasp back to a cohort with an almost double so long follow-up period including patients with late recurrence 5 years after the initial diagnosis (Table 1). Considering further the intersecting arms in the survival curves displayed in Figure 2J, we analyzed whether hsa-miR-516-3p expression may be time dependent using the Schoenfeld residual test [21]. The test revealed that the proportional hazards assumption was significantly violated with respect to DFS (*p* = 0.0012) and OS (*p* = 0.001) (Figure 3A and 3C), thus precluding the application of the COX regression model [21, 22]. To account for non-proportionality we conducted a landmark analysis using two time intervals (Figure 3B and 3D). The intervals were defined based on the time points at which the hazard ratio assessed by the Schoenfeld residuals changes from above one to below one. For DFS and OS an even more significant association of low hsa-miR-516-3p expression with an unfavorable prognosis was determined 3.35 (HR:0.15, *p* = 0.0018) and 4.5 years (HR:0.06, *p* = 0.00030) post diagnosis, respectively (Figure 3B and 3D). However, no significant association could be detected in the first time interval although a trend was observed pointing in the opposite direction. In summary, our results contradict the initial findings by Foekens *et al.* but may point to a time-dependent prognostic role of hsa-miR-516-3p.

## DISCUSSION

A general challenge for the identification and validation of prognostic miR markers is the suspected low comparability between standalone studies. Potential prognostic candidates are individually identified dependent on the quality of RNA and clinical data, patient characteristics as well as the selected study design and methodology. Stringent statistical filtering and correction for multiple testing often limits the number of assigned positive hits. As a result, the majority of miRs are classified as negative hits although one cannot rule out that a number of promising candidates may have been filtered out. Other studies may identify these as promising candidates such that different standalone studies rarely identify overlapping candidate sets [12]. Although, subsequent validation in independent cohorts is often applied to confirm the prognostic potential of a positive candidate, unfortunately, the confirmation rate is likely to be low because of the aforementioned heterogeneity between studies. However, the successful reproduction of results for a positive hit is indeed expected to have increased explanatory power, because it is very unlikely that the same biomarker shows significant results in two truly independent cohorts just by chance.

We evaluated the prognostic potential of seven previously proposed miRs in systemically untreated breast cancer patients independent of classical clinical and pathological marker. Besides analyzing publicly available data sets we introduce our new data set comprising 110 paired primary tumors of systemically untreated patients with very long follow up time. Matching of the samples minimized bias concerning clinical and pathological characteristics as well as bias related to storage time, sampling method, and diagnostic procedures. Consequently, the chosen paired design increased the power of our analysis in comparison to cohort studies by omitting any effect potentially caused by an unbalanced cohort composition and by enriching for relevant clinical endpoints [18].

We found an association of hsa-miR-548c-5p with outcome in our data set supporting previously published results. For the remaining candidate miRs no significant validation results were obtained. A lack of power due to the lower number of samples analyzed in our study may cause the bias. However, the use of untreated samples with prolonged follow up was a prerequisite to search for potential marker which can identify patients who may not benefit from any systemic treatment.

In comparison, the re-analysis of published data supported a prognostic role of hsa-miR-30e-3p, hsa-miR-7-5p, hsa-miR-210-3p and hsa-miR-128-3p. However, none of the analyzed candidate miRs could be confirmed as being prognostic in all four tested cohorts. For two miRs, significant results were only computed in a single cohort. Although we aimed at finding best matching cohorts, we were limited to systemically untreated samples of LN negative and ER positive patients. Moreover, the comparability of the included cohorts may not be optimal considering e.g. that only marginal clinical data were available for the Foekens *et al*. cohort or, that the Metabric cohort was quite heterogeneous. Furthermore, in all previously published studies considerable shorter follow-up times were available. These differences as well as variances in sample handling and differences in the miR purification protocol may have reduced the reproducibility across different studies. Result bias may further be caused by varying platform performance considering reproducibility, accuracy, detection rate, sensitivity and specificity [20, 23, 24]. Foekens *et al*. quantified miRs using a qPCR-based approach (TaqMan, Applied Biosystems) while D’Aiuto *et al*. used MicroRNA expression beadchips from Illumina. Interestingly, results for three out of four miRs identified by Foekens *et al.* were validated using the D’Aiuto *et al*. data set potentially indicating an improved comparability between technologies including PCR amplification steps. In comparison, data collected based on miR hybridization on customized arrays produced by Agilent (Metabric) and on our own arrays based on a probe library provided by Exiqon verified results for three of the seven suggested candidates. Mestdagh *et al.* analyzed the differential expression of two identical sample groups via 12 platforms (applying qPCR, hybridization or sequencing for analysis) and detected 66 differentially expressed miRs by at least one platform [20]. However, only two of these miRs were concordantly detected as differentially expressed by all platforms [20]. Thus, platform-dependent false positive and negative hits are likely to occur and the careful selection of similar performing platforms may be critical for a robust validation of candidate miRs when using different cohorts.

Previous studies further attempted to causally link the seven miR candidates to specific functions in breast cancer progression. For example, D’Aiuto *et al.* reported that the prognostic hsa-miR-30e-3p is functionally linked to the induction of epithelial-mesenchymal-transition (EMT) which is a prerequisite progress for tumor cell dissemination [14]. This result is in line with previous studies suggesting a critical role of the miR-30-family in EMT [25, 26]. We found high hsa-miR-548c-5p expression significantly associated with abridged disease free-survival. The prognostic role of this miR may even be independent of the receptor status or treatment, because Boukerroucha *et al.* identified a predictive role of hsa-miR-548c-5p in conjunction with three other clinic-histopathological parameters in triple negative breast cancer (TNBC) [27]. On the contrary, functional studies in endometrial, ovarian and liver cancer suggest that decreased hsa-miR-548c expression promotes cell migration and invasion potentially by silencing the EMT marker TWIST [28, 29]. Accordingly, further studies in ER positive breast cancers are required to verify the functional and prognostic role of these miRs.

In comparison, miR-7 was shown to function as breast cancer invasion and metastasis inhibitor and to mediate cytotoxic T-lymphocyte-mediated lysis of breast cancer cells [10, 30]. Moreover, miR-7 was shown to suppress cell proliferation and induce apoptosis in different breast cancer cell lines [31]. Conversely, Foekens *et al.* associated a high expression of hsa-miR-7-5p with abridged disease free survival and; we found hsa-miR-7-5p significantly upregulated in metastasizing tumors of the D’Aiuto *et al.* cohort. Differences in receptor status, strand selection and endogenous expression may compromise comparability of these studies and may explain varying functional roles for miR-7 dependent on the examined cellular background.

MiR-210 has also extensively been analyzed in breast cancer and several studies associated miR-210 expression with breast cancer survival [9, 10, 12, 32]. However, we could not confirm the prognostic role of hsa-miR-210-3p in primary tumor samples although a significant differential expression was detected in local versus disseminating primary tumors [33]. Wang *et al.* performed a systematic meta-analysis of miR-210 in different cancer types and found no significant association with breast cancer [34]. Contradicting results were reported by Xie *et al.* and Hong *et al.* in two meta-analysis studies [35, 36]. However, all studies included mixed patient cohorts including treated patients and varying receptor status. The same is true for functional studies which linked miR-210 to treatment resistance, hypoxia and a cancer promoting role in ER negative breast cancers [10, 32].

Aberrant expression of miR-128 was reported in many different malignancies including e.g. breast, colon, lung, brain and pancreatic tumors [37, 38]. Buffa *et al.* significantly associated high hsa-miR-128a with reduced distant relapse free-survival in ER positive breast cancer samples receiving systemic adjuvant treatments [33]. They further confirmed their results via an integrative meta-analysis and Lánczky *et al.* independently validated the predictive role of miR-128a [12, 33]. These findings are in good agreement with our results. However, these studies included treated patients and Masri *et al.* associated high miR-128 expression with letrozole resistance in breast cancer [39]. Consequently, the results may suggest that miR-128a may promote breast cancer recurrence by not only promoting metastasis formation but also by mediating treatment resistances.

Surprisingly, hsa-miR-125b-5p was neither differentially expressed nor significantly associated with survival in our study, although a tumor suppressive role of miR-125b was recurrently reported in cell line models [40, 41]. Moreover, miR-125b is part of a 10-miR classifier which significantly predicts 5-year distant relapse free survival in endocrine treated, hormone receptor positive and ERBB2 negative breast cancer patients [42]. In summary, miR-125b-5p may positively support endocrine treatments but may not be causal for the occurrence of metastases.

In comparison to the other analyzed miRs, little is known about the functional role of miR-516a. White *et al.* reported, that miR-516a is one out of three miRs regulating kallikrein 10 expression and cell proliferation in breast cancer [43]. The database Tarbase (http://www.microrna.gr/tarbase) proposes sulfatase 1 (*SULF1*) as the only validated gene target of hsa-miR-516a-3p [44]. SULF1 was reported to be a tumor suppressor, and to be downregulated in primary breast cancer tissue [45]. Correspondingly, an upregulation of hsa-miR-516a-3p is assumed to reduce SULF1 and the positive effects mediated by the gene which is contradicting our findings, that a low hsa-miR-516a-3p is associated with unfavorable outcome. However, our findings oppose the initial outcome association published by Foekens *et al.* [13]. Unfortunately, no patient specific expression data and clinical and pathological information were available for the Foekens *et al.* cohort which would allow us to re-analyze the data and to potentially point out causal difference in the cohort composition which may explain contradicting results. While this miR was not included in the Metabric study, an analysis in the independent D’Aiuto *et al.* cohort did not significantly indicate a beneficial up- or downregulation of the miR, although we observed a slight trend towards an advantageous high expression. However, follow up of patients was much longer in our study. Furthermore, we had to divide our data into two time intervals to not violate the proportional hazards assumption of the COX model. We observed, that high expression is potentially associated with a worse prognosis within the first years after diagnosis (3.35 and 4.5 years for DFS and OS, respectively), while thereafter high expression is significantly associated with prolonged survival. It is acknowledged, that the Foekens *et al.* cohort includes almost double as many patients as our cohort. However, by using the paired design we increased the prognostic power for our analysis as discussed above.

In conclusion, using data of four independent cohorts analyzed by different analysis platforms we collected further indications supporting the prognostic potential of hsa-miR-548c-5p, hsa-miR-7-5p, hsa-miR-210-3p and hsa-miR-128-3p. In addition, a potentially time-dependent role was determined for hsa-miR-516a-3p. We exclusively selected systemically untreated, LN negative and ER positive breast cancer patients for a treatment independent analysis. The stringent selection limited the number of patients to be analyzed and may reduce the prognostic power of the study, however, only this particular patient group is suitable to identify miRs which can better classify low-risk breast cancer patients and thus have the potential as clinical marker to reduce overtreatment in the future. Nevertheless, further studies in large, homogeneous cohorts with standardized sample treatment and long follow up using accurate and selective platforms are required to eventually confirm the prognostic potential of these candidates for clinical use.

## MATERIALS AND METHODS

### Tumor biopsies

Frozen tumor biopsies were collected from 110 lymph node negative and ER positive (measured by gene expression) patients who were diagnosed with breast cancer between 1980 and 1999 on the island of Funen, Denmark. All patients underwent surgery to remove the primary tumor, but none of the patients received systemic neoadjuvant or adjuvant therapy. Pathological examination of snap-frozen tumor samples was performed at the Department of Pathology at the Odense University Hospital, Denmark which confirmed a tumor cell content of more than 50% in all cases. Fifty-five patients developed metastasis within 10 years after diagnosis, while 55 patients did not experience metastasis (followed for at least 8 years; mean follow up 19.6 years). Patient biopsies were matched according to tumor type, year of surgery, tumor diameter (range: 6–50 mm), age (range: 33–88 years), receptor status (ER, PR, n/a) and histological grade (grade 1–3 or n/a) (Table 1). All clinic-pathological information was extracted from the Danish Breast Cancer Cooperative Group (DBCG) database, the Funen pathology database, or the nationwide pathology database. The study was approved by the Danish National Committee on Health Research (S-VF-20020142). The study was retrospective and no informed consent was obtained from the patients involved in the study as approved by the Ethical Committee.

### RNA extraction

Total RNA was isolated from the freshly frozen tumor biopsies with Trizol (Invitrogen) and further purified with the RNeasy micro kit (Qiagen), including DNase treatment. NanoDrop Spectrophotometer (NanoDrop Technologies) was used for RNA quantification. The quality of extracted RNA was assessed with Bioanalyzer 2100 (Agilent Technologies) using the RNA 6000 Nano Kit (Agilent Technologies).

### Microarray quantification and analysis

The miRCURY LNA microRNA array ready-to-spot probe-set was purchased from Exiqon. The set contains capture-probes for 1212 human miRs based on the miRBase version 16 (http://www.miRbase.org/) [19]. Briefly, the probes were spotted in triplets on CodeLink HD (SurModics) activated glass slides and post-processed according to the CodeLink protocol. The miRCURY Power labeling kit (Exiqon), miRCURY LNA Array hybridization buffer (Exiqon) and miRCURY LNA Array Washing buffer kit (Exiqon) were used for sample labelling with Hy3, hybridization and washing, respectively. Equal amounts of RNA from all included samples were pooled and used as common reference labeled with Hy5. Hybridization, washing, and scanning (Agilent G2565CA Microarray scanner) were performed according to the recommendations provided by Exiqon.

Scanned images were imported into GenePixPro6.0 software (Molecular Devices) for quality control and raw data extraction. The R-package *limma* was used for LOESS and quantile normalization of raw signal intensities, and the ComBat function embedded in the *sva* R-package was used for adjustment of the normalized intensities and to eliminate potential batch effects [46, 47]. Gene expression was initially defined as the ratio between Hy3 and Hy5 signal intensities. For the purpose of cross dataset miR expression comparability we further standardized these calculated ratios and used these values for differential expression and survival analysis. Microarray data have been deposited in NCBI’s Gene Expression Omnibus [48] and are accessible through GEO Series accession number GSE103161 (https://www.ncbi.nlm.nih.gov/geo/query/acc.cgi?acc=GSE103161).

### Validation data acquisition

Publicly available miR expression data published by D’Aiuto *et al.* were collected from NCBI’s Gene Expression Omnibus database (acc=GSE59829) [48]. Expression data published by Dvinge *et al.* and Curtis *et al.* (Metabric) were retrieved from the European Phenome-Genome Archive (https://www.ebi.ac.uk/ega/home) after access was granted by the Metabric consortium [15, 16, 49]. For consistent miR nomenclature the R-package *miRNAmeConverter* was applied and MIMAT numbers were used as identifier in subsequent analysis [50].

### Statistical analysis

Student’s *t*-tests were performed to analyze differential expression. For each of the seven miRs we divided the samples into groups with high and low expression using the median expression as cut off. The grouping was used for subsequent analyses using the COX univariable proportional hazard model [22].

Overall survival (OS) was defined from diagnostic date to death or loss of follow-up. Disease free survival (DFS) was defined from diagnostic date to metastasis recurrence or loss of follow-up. The Kaplan–Meier method was applied to describe the OS and DFS. Significant discrepancies of OS or DFS were assessed by univariable COX proportional hazard analysis [22, 51]. The proportional hazards assumption and time-dependent effects were examined by plotting the scaled Schoenfeld residuals to test for significant deviation from a zero slope, respectively [21]. Landmark analyses were applied to assess the hazard ratios during particular periods. The landmarks were defined as the time point upon which the Schoenfeld assessed Hazard Ratio (HR) shifts from >1 to <1, thus being (0–3.35 years and >3.35 years for DFS) and (0–4.5 years and >4.5 years for OS). This analysis therefore included patients who were event free at the start of each period, and being censored at the end of each period. All survival analyses were conducted using the *survival* R-package [22, 51]. All *p*-values below 0.05 were considered statistically significant.

## Abbreviations

OS: Overall survival
DFS: Disease free survival
ER: estrogen receptor
LN: lymph node
PR: progesterone receptor
ERBB2: erb-b2 receptor tyrosine kinase 2
miR: microRNA
Metabric: Molecular Taxonomy of Breast Cancer International Consortium
DBCG: Danish Breast Cancer Cooperative Group
OUH: Odense University Hospital
Std. expression: Standardized expression
HR: Hazard ratio
CI: Confidence interval
STDEV: Standard deviation.
